# Editorial: Epigenetic and metabolic regulators of breast carcinogenesis, volume II

**DOI:** 10.3389/fonc.2025.1726066

**Published:** 2025-11-27

**Authors:** Maha Saber-Ayad, Noha Mousaad Elemam, Iman Mamdouh Talaat, Hauke Busch

**Affiliations:** 1 Department of Clinical Sciences, College of Medicine, University of Sharjah, Sharjah, United Arab Emirates; 2 Research Institute for Medical and Health Sciences, University of Sharjah, Sharjah, United Arab Emirates; 3 Division for Medical Systems Biology, Lübeck Institute of Experimental Dermatology, Universität zu Lübeck, Lübeck, Germany; 4 University Cancer Center Schleswig-Holstein, University Hospital Schleswig-Holstein, Lübeck, Germany

**Keywords:** breast cancer, CHD4, microRNAs, coagulation markers, tumor microenvironment, metabolic reprogramming, epigenetic regulation

## 1 Introduction

Breast cancer (BC) is the most prevalent cancer among women worldwide, characterized by key features including epigenetic dysregulation and metabolic reprogramming. Epigenetic mechanisms, including histone modification, DNA methylation, and non-coding RNAs, dynamically regulate oncogene and tumor suppressor gene expression, influencing carcinogenesis and the tumor microenvironment. Meanwhile, metabolic reprogramming supports tumor initiation, progression, invasion, and metastasis, allowing cancer cells to adapt to challenges like nutrient deprivation, hypoxia, and chemotherapy. Advances in BC research have revealed significant tumor heterogeneity, attributed at least in part to epigenetic modifications, and novel metabolic pathways driving malignancy.

Triple-negative BC (TNBC) remains one of the most aggressive and difficult-to-treat subtypes due to the absence of ER, PR, and HER2 receptors. With limited therapeutic options, epigenetic mechanisms have emerged as promising targets. The review by Mahendran et al. highlighted the roles of DNA methylation, histone modifications, and microRNA (miRNA) regulation in the pathogenesis and therapy of TNBC. DNA methylation inhibitors aim to restore tumor suppressor activity, while histone deacetylase inhibitors (HDACi) show potential in reversing aberrant gene silencing, particularly when combined with kinase inhibitors. Meanwhile, miRNA-based therapeutics offer dual roles as tumor suppressors or oncogenes. Together, these strategies may pave the way for more effective, personalized treatments for TNBC.

Chromatin domain-binding protein 4 (CHD4), the ATPase core of the NuRD complex, plays a dual role in epigenetic regulation by mediating both gene activation and repression. Dong et al. described a case of occult BC with extensive bone metastasis harboring a novel truncating somatic mutation in the catalytic SNF2 domain (CHD4 p.Trp736Ter). This alteration, not previously reported in any cancer, disrupts ATPase function and destabilizes NuRD complex formation. A comprehensive multimodal evaluation, including PET/CT imaging, biomarker profiling, and pathology, confirmed the luminal-A subtype and a significant response to endocrine therapy. They suggested that this CHD4 mutation may alter its regulatory functions, boosting gene activation while impairing chromatin remodeling. Such dysfunction could promote metastatic progression through abnormal transcriptional control, indicating a possible mechanistic link between CHD4 loss-of-function mutations and tumor spread in hormone receptor-positive BC.

Metabolic risks (MRs) are significant contributors to BC mortality among women. Using data from the Global Burden of Disease Study 2019, the study by Zhang et al. examined trends over time and the effects of age, period, and cohort on MR-related BC mortality in Chinese women aged 25 and older. Joinpoint regression showed that the age-standardized mortality rate (ASMR) increased from 1990 to 2019, with an average annual percentage change (AAPC) of 1.79% (95% CI: 1.69–1.87). Mortality linked to high fasting plasma glucose (HFPG) and high body mass index (HBMI) increased by 0.41% and 2.75%, respectively. Age-period-cohort analysis indicated that BC mortality related to HBMI increased significantly with age, especially in women over 50, and surpassed the impact of HFPG. The risk associated with HBMI has steadily grown since 2005, while HFPG showed a temporary rise followed by a decline. Therefore, middle-aged and elderly women should be prioritized for HBMI and HFPG management to reduce BC mortality.

The study by Lu et al. extends the metabolic perspective of breast carcinogenesis by highlighting the link between coagulation activity and tumor metabolism. Cancer-associated changes in coagulation reflect underlying metabolic and epigenetic reprogramming within the tumor microenvironment. Thus, the prognostic value of the APTT/TT ratio may serve as a surrogate marker of broader metabolic adaptations driving BC progression. This retrospective study evaluated whether coagulation markers predict outcomes in 264 women with non-metastatic BC treated with surgery and adjuvant therapy at Suqian Hospital, China. Focusing on the APTT/TT ratio (activated partial thromboplastin time to thrombin time), the authors identified 1.4 as the optimal cut-off for predicting 5-year disease-free survival (DFS). Patients with APTT/TT ≥ 1.4 had significantly shorter DFS. A multivariate Cox analysis elevated APTT/TT (Hazard Ratio (HR) ≈4.1) and lymph-node metastases (HR≈2.3) were independent adverse prognostic factors, whereas tumor size lost significance. The study concludes that APTT/TT is a low-cost, accessible biomarker that, combined with lymph-node status, improves risk stratification in BC after comprehensive therapy, though validation in larger prospective cohorts is needed. Although the study primarily focuses on coagulation dynamics, it demonstrates cancer-associated hypercoagulability, as a result of a broader reprogramming of cellular energy balance, inflammation, and the tumor microenvironment.

A meta-analysis by Gonzalo-Encabo et al. assessed the prognostic value of a calculated Systemic Immune-Inflammation Index (SII) in patients with BC. Chronic inflammation and immune dysregulation are known to influence tumor progression, but SII’s predictive role in BC outcomes remained uncertain. The authors pooled data from 11 retrospective cohort studies involving more than 6,000 patients. Outcomes included overall survival (OS) and disease-free or progression-free survival (DFS/PFS). Using random-effects models, the meta-analysis showed that elevated SII was consistently associated with worse OS (HR ≈ 1.9) and shorter DFS/PFS (HR ≈ 1.7) compared with low SII levels. Subgroup analyses confirmed these findings across geographic regions, SII cut-offs, and treatment modalities. The study concludes that SII, as an independent predictor of poor prognosis, has potential as a low-cost, readily available biomarker to complement established clinicopathologic factors.

The bibliometric analysis by Yang et al. of over 5,200 studies (2000–2024) maps the evolution of the field of epigenetic research in BC, from early work on promoter hypermethylation of tumor suppressor genes to recent explorations of multi-omics integration, tumor microenvironment interactions, and synthetic lethality. They identified that the United States and China dominate research output, with major contributions from institutions, like Johns Hopkins and MD Anderson. Four research phases were identified, spanning gene methylation studies, mechanistic insights, translational biomarker and drug development, and recent multi-omics integration. Emerging priorities include targeting epigenetic memory, metabolism-epigenetics crosstalk, and single-cell profiling, offering a roadmap for future therapeutic innovation in BC.

## 2 Conclusion

The collective findings from these studies have shed light on multiple epigenetic and metabolic pathways underlying breast carcinogenesis. Altogether, this body of work deepens current insights into BC pathogenesis and inspires innovative approaches to enhance diagnostics, prognostics, and ultimately, patient outcomes in this vital field of oncology.

